# Clinical Test of a Wearable, High DOF, Spring Powered Hand Exoskeleton (HandSOME II)

**DOI:** 10.1109/TNSRE.2021.3110201

**Published:** 2021-09-16

**Authors:** Rafael Casas, Melissa Sandison, Tianyao Chen, Peter S. Lum

**Affiliations:** Biomedical Department, The Catholic University of America, Washington, DC 20064 USA

**Keywords:** Exoskeleton, stroke, movement therapy, hand

## Abstract

In previous work, we developed an exoskeleton, Hand Spring Operated Movement Enhancer (HandSOME II), that allows movement at 15 hand degrees of freedom (DOF). Eleven separate elastic elements can be added to customize the extension assistance for individuals with impaired hand function. In this pilot study of twelve individuals with stroke, we measured the immediate improvements in range of motion (ROM) and upper extremity function when wearing the device. Index finger ROM was significantly improved at the PIP (p=.01) and DIP joints (p=.026), and the max extension was significantly increased at the MCP (p<.001), PIP (p=.013) and DIP joints (p=.016). The thumb CMC abduction max (p=.017) and CMC flexion/extension ROM also increased (p=.04). In a grip and release task involving various objects, six subjects were unable to complete the tasks without assistance. Across these 6 subjects, 13 of 42 tasks were completed without assistance, while 36 of 42 tasks were completed when wearing HandSOME II. Despite the extension assistance provided by the device, flexion grip force was not statistically decreased. HandSOME II can potentially increase the effectiveness of repetitive task practice in patients with moderate-severe hand impairment by allowing completion of grasp and release tasks that are impossible to complete unassisted.

## I. Introduction

**T**HERE are approximately 800,000 new stroke cases every year in the US [1]. Fifty percent of affected individuals over the age of 64 show hemiparesis at six months and 26% of them are fully dependent on others for activities of daily living (ADL) [2]. Rehabilitation in the upper extremity targets reaching and grasping movements, but in order to use the upper limb for ADL, a high degree of motor control is needed. To complicate matters, hand motor control is commonly impaired and the odds of regaining function are low [3]. Current therapy guidelines are to promote functional training of ADL for the affected arm and specialized task-oriented approaches that rely on repetitive practice [4].

Motor recovery of the paretic hand is a critical component for functional recovery in the upper limb [5]. Many stroke survivors have some degree of voluntary finger flexion; however, finger extension is limited by inappropriate activity in flexors [6] and inability to activate extensors [7]. By applying extension force to the fingers, exoskeletons can assist the effectiveness of task-related repetitive practice by enabling completion of their movements, increasing the effectiveness of task practice in the upper extremities [8]. A recent meta-analysis found that robotic devices provide larger gains in arm function, strength and ADL ability in comparison to other interventions [9]. Unfortunately, patients have limited access to robotic interventions because they are often too expensive for most clinics or individuals to purchase. In addition, most devices are not wearable for use during ADL, which limits transfer of gains to real world everyday use of the upper extremity [10].

Powered hand exoskeletons that can be integrated into ADL practice have shown success in improving hand function of stroke patients [11]–[14]. However the electronics, motors or pneumatic power sources of these devices result in increased weight, complexity and cost. Passive devices utilize spring or elastic elements for actuation, decreasing weight and eliminating the need for any electronic components and external power sources. In the Saebo Flex, metal springs are connected to the distal phalanx of each finger and can assist with hand opening and tone management therapy [15]. Due to the increasing extension force as the fingers are flexed, achieving full active range-of-motion is usually not possible and grasping small objects is difficult. Additionally, assistance is only provided for the proximal interphalangeal (PIP) joints, with movement at the distal interphalangeal (DIP) and metacarpophalangeal (MCP) joints restricted. In the Saebo Glove, elastic bands provide extension assistance at the MCP and PIP joints, however this device cannot produce enough torque for patients with severe or moderate impairments. Another passive device, SCRIPT, uses Saebo Flex components and achieves greater finger ROM by applying external extension torques with a combination of passive leaf springs and elastic tension cords [16].

Our lab previously designed and tested the lightweight, passive, and portable Hand Spring Operated Movement Enhancer (HandSOME I) device that was designed for pinch-pad grasp by bringing the pads of the thumb and fingers together. This device assists with ADL practice of grasping tasks, but is designed as a neuro-rehabilitation retraining device. The device utilizes elastic cords for passive actuation of extensors. Its design assists finger and thumb coordination through a linkage that couples together movements of these digits. Studies showed improved ROM and functional grasp by individuals with stroke [17], [18]. A home training study found significant gains in hand function after 4 weeks of daily training [18].

**Fig. 1.**
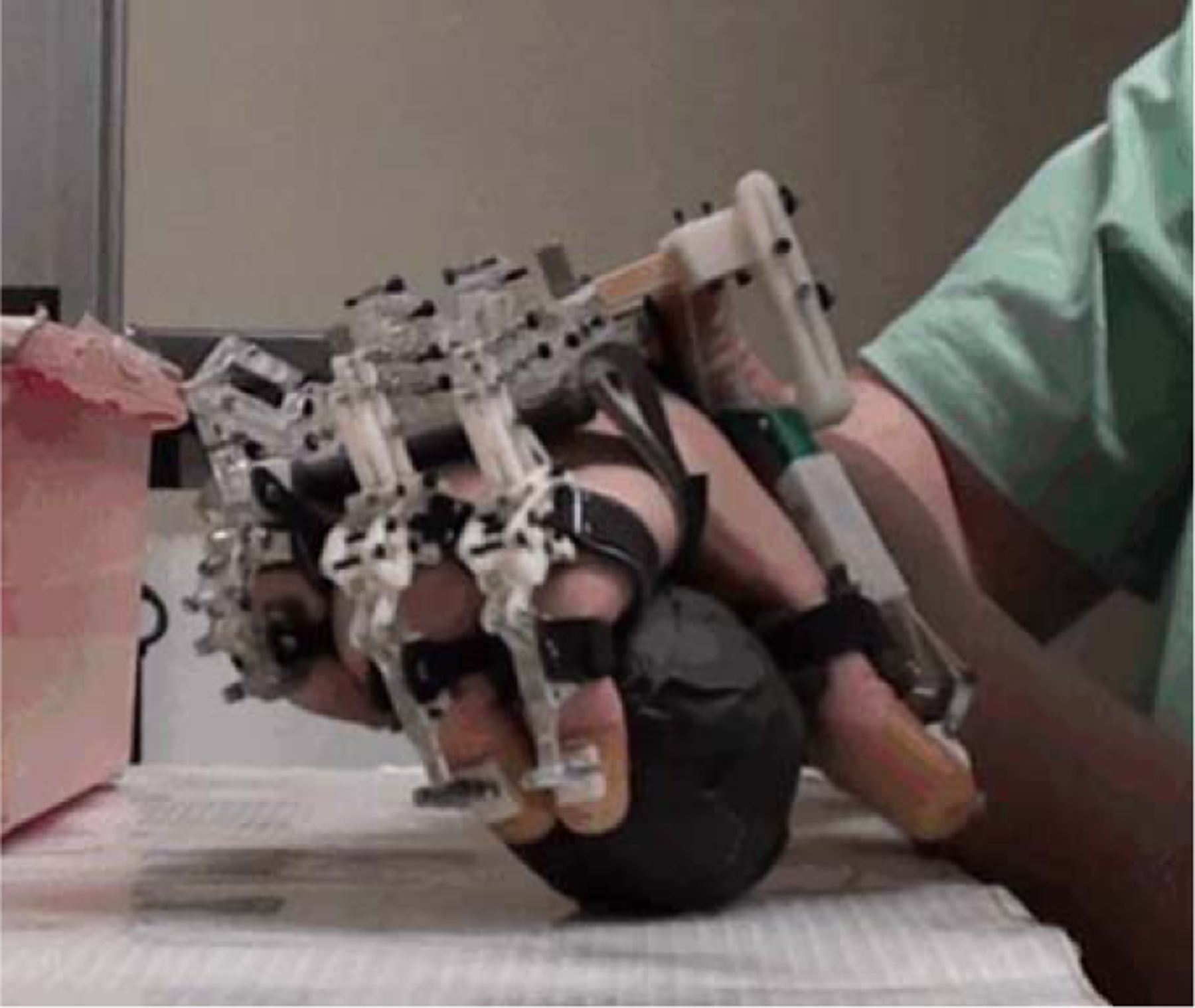
HandSOME II device providing extension assistance in order to grasp object.

**Fig. 2.**
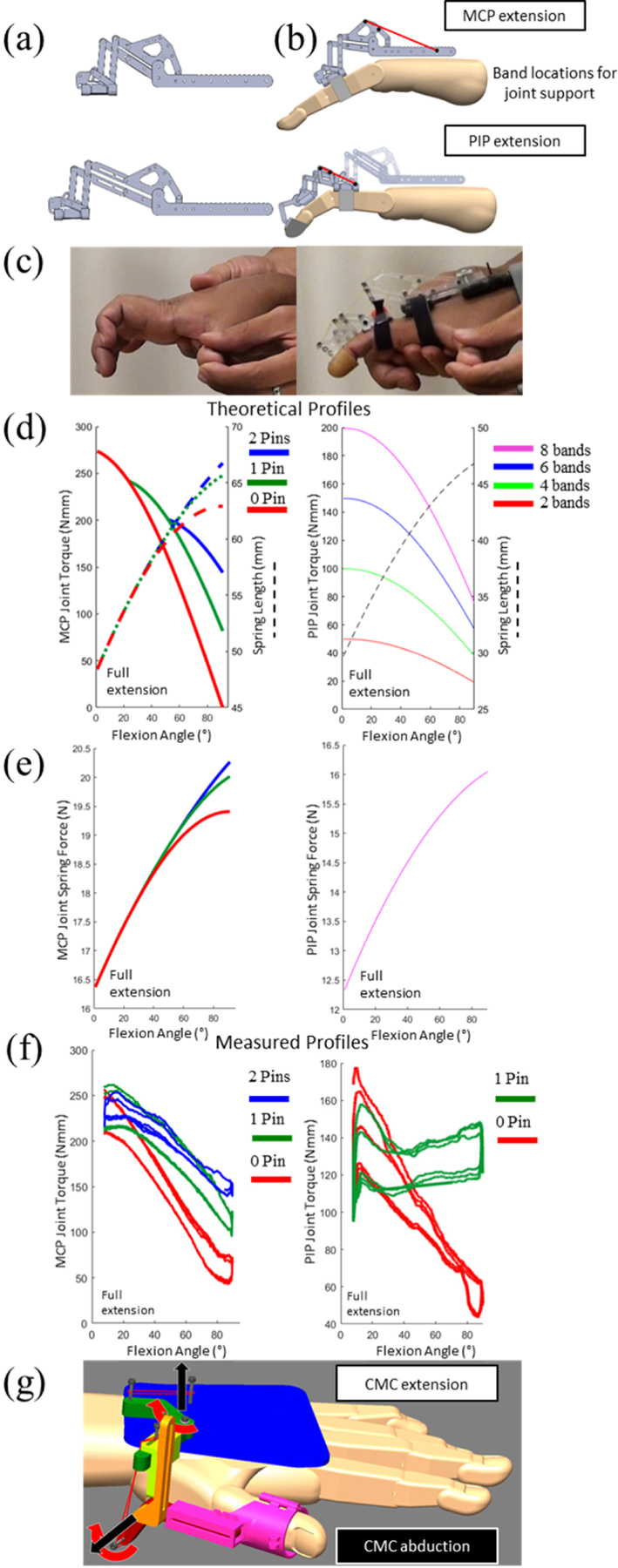
(a) Linkages can be sized based on finger length (b) HandSOME II device with location of rubber bands (red lines) at the MCP and PIP joints. (c) Abnormal finger posture in one subject that is improved when wearing HandSOME II. (d) (Left) Theoretical MCP torque profiles with different spring path options. Full extension is 0 degrees and the x-axis represents flexion angle. 8 rubber bands were used. Dashed lines represent theoretical spring length at the MCP. (Right) Calculated PIP torque profiles with “1 Pin” spring path option. Lines represent different rubber band count. Dashed line represent theoretical spring length at the PIP. (e) (Left) Calculated MCP spring force profile (Right) Calculated PIP spring force profile (f) (Left) Measured MCP torque profile with different spring path options using 8 rubber bands. (Right) Measured PIP torque profile with 4 rubber bands. (g) Thumb piece of HandSOME II device supporting CMC extension and CMC abduction.

The HandSOME II expands the HandSOME concept to control more complex grasp patterns used in ADL [19] (Fig. 1). The HandSOME II is comprised of rigid mechanical linkages that allow isolated joint movement and elastic elements that provide customized extension assistance for each finger and thumb DOF. Each finger exoskeleton is strapped to the finger segments, and located on the back of the hand to avoid interference between fingers at a closed hand position. The linkages achieve centers of rotation aligned with the finger MCP and PIP joints to maximize comfort and proper kinematics. Linkages are sized and adapted to subject’s finger size (Fig. 2(a)). As seen in Fig. 2(b) and Fig. 2(c), adjustable elastic bands provide extension torque at the MCP and PIP joints. The device retains the characteristic used in HandSOME I of an adjustable assistance profile that decreases or remains constant as the flexion occurs. The amplitude of assistance is adjusted by adding rubber bands in parallel. The shape of the torque vs. angle assistance profile can be adjusted by use of optional wrapping “pins” that alter the path of the rubber bands and increase the torque in the fully flexed position (Fig. 2(d)). These pins are useful in cases where the rubber bands provide enough torque to hold the finger in full extension, but the fingers cannot be opened after a grasping movement. Rubber band stiffness was measured and found to be linear over the operating range (.028 N/mm). This was used to calculate force profiles (Fig. 2(e)). Through benchtop testing, torque profiles were measured directly for MCP and PIP joint (Fig. 2(f)). Movement at the PIP and DIP are coupled together through a gear since they normally move synchronously. For the thumb (Fig. 2(g)), the carpometacarpal (CMC) joint has three DOFS: abd/adduction, flex/extension and oppo/reposition. The mechanical design has DOFs for abd/adduction and flex/extension. Separate elastic bands were used to support CMC abduction, CMC extension and interphalangeal (IP) extension. All parts were laser cut acrylic plastic or 3D printed ABS plastic. Full details on the HandSOME II mechanical design have been reported previously [19].

While the technical description of the device has already been reported, the goal of this study was to examine how much the HandSOME II device improves range of motion (ROM) and increases success rate in reach and grasp tasks in individuals with various levels of hand impairment due to stroke. This study is the first description of clinical testing with this novel device and would provide guidance on future clinical training studies.

## II. Methods (Clinical Testing)

Twelve chronic stroke patients (7 males/5 females, mean age 59 ± 13.6 years, 33 ± 18.8 months post stroke onset) participated in this study. We recruited patients with a wide range of upper limb Fugl-Meyer assessment (FMA) [20] scores (range 15–47, mean 32) to evaluate the performance of the device on differing levels of motor impairment post stroke. Patients were excluded from the study if they did not have the ability to volitionally close their hand after it had been passively opened by the screening occupational therapist. Informed consent was provided by all the participants. Study protocol was approved by the MedStar Health Research Institute Institutional Review Board (#2012–011).

### A. Testing Procedure

A motion capture system was used to record index finger and thumb movements for range of motion analysis (Osprey, Motion Analysis Corporation, Santa Rosa CA, USA). Spherical tracking markers were placed directly on the MCP, PIP, DIP and finger tip of the index finger. On the thumb, markers are placed at the IP, MCP, CMC and thumb tip. Additional markers were placed on the back of the hand to define the reference plane for measuring index finger and thumb joint angles. The other fingers were not tracked, as pilot testing found it was impossible to track their movements due to marker occlusion from the exoskeleton. Thumb CMC extension and abduction angles were calculated relative to the fully flexed and adducted position of the joint. Index finger extension angles of the DIP, PIP, and MCP joints were calculated assuming full extension was 180°. The 0° flexion angle was assigned to full extension. During the range of motion trials, the forearm was pronated and supported on a cushioned surface that crossed the wrist into the palm, so as to support the wrist in neutral. The surface was elevated enough to allow a comfortable horizontal position for the forearm to rest on the table and to allow the fingers to move without interference from the support or table. In a time frame of 30 seconds, subjects were asked to make three attempts of closing and opening their hand as far as possible to determine range of motion of the index finger and thumb. In the initial 4 subjects, thumb markers were occluded. In the rest of the subjects, thumb ROM data was collected separately in another 30 sec trial after the subject’s hand was repositioned to ensure the thumb markers were captured.

Additionally, subjects were required to complete 7 picking and place tasks designed to require a range of grasp patterns. For each task, subjects were seated and allotted 30 seconds and unlimited number of attempts to perform one successful trial of picking up a daily object on the table with their impaired hand, and dropping it into a bin on the table. The bin was 5.3 inches tall and placed directly in front of the object to reduce the amount of arm movement required. If the object was dropped, the object was immediately repositioned for the subject to try again, until the 30 seconds was over. The seven daily objects include: 1 inch wood block, 2 inch wood block, 3 inch wood block, baseball, marble, pen, and key. The first 5 objects were based on objects used in the Action Research Arm Test [21]. The pen was added as another pinch task using a more commonly used object (as compared to the marble). The key was added to test for key-pinch grasp. During the pick and place tasks, there was no support provided for the wrist. While the back brace of the device does extend across the wrist, the proximal strap was not used, as we thought a wrist support alone might result in improved performance. The subject held the affected arm with the non-affected hand at the distal portion of the forearm and provided assistance to positioning and lifting the affected arm as needed during the pick and place tasks. Subjects completed all tasks without the device initially, then with the device following a rest period. We recorded the number of successes and the amount of time needed to complete the task for each patient. After all tasks were completed, grip force was measured with and without the device using the Jamar Dynamometer.

### B. Selecting Ideal Spring Settings and ROM

The ideal number of elastic bands varied between subjects. In order to best match the subject’s tone profile for each joint in the fingers, the patient was asked to extend their hand in the device. Rubber bands were added until each finger was in full extension. The wrapping pins were used when a subject could close the hand from an open position, but could not reopen. The same procedure was done with all the finger and thumb joints.

### C. Kinematic Outcomes and Statistical Testing

For the biomechanical evaluation of the HandSOME II device, we focused on kinematics of the metacarpophalangeal (MCP), proximal interphalangeal (PIP), and distal interphalangeal (DIP) joints of the index finger; specifically flexion/extension and ROM. For the thumb, we analyzed flexion/extension and ROM of the carpometacarpal (CMC) and distal interphalangeal of the thumb joint (tDIP), and abduction/adduction and ROM of the CMC.

Compound index finger flexion and extension was computed as the mean of MCP, DIP and PIP joints (EXT_FINGER_ and FLEX_FINGER_). Compound index finger ROM (ROM_FINGER_) is maximum EXT_FINGER_–maximum FLEX_FINGER_.

Compound thumb opening motion was computed by the mean of CMC abduction, CMC extension and tDIP extension (EXT_THUMB_), while compound thumb closing motion was the mean of CMC adduction, CMC flexion and tDIP flexion (FLEX_THUMB_). Compound thumb ROM is maximum EXT_THUMB_–maximum FLEX_THUMB_.

To calculate a measure of total hand opening movement (EXT_HAND_), we averaged EXT_FINGER_ and EXT_THUMB_, while total hand closing motion (FLEX_HAND_) is the average of FLEX_FINGER_ and FLEX_THUMB_. Total hand ROM (ROM_HAND_) is maximum EXT_HAND_– maximum FLEX_HAND_.

Velocity was calculated using the most distal marker of the thumb and index finger (VELOCITY_FINGER_ and VELOCITY_THUMB_). First we computed the separate X, Y and Z velocities by taking the derivative of the position data. The derivatives at the first and last points were calculated by the 3 point forward and 3 point backward finite difference scheme respectively. The derivatives at all the other points are calculated by the 2 point central difference approach. Tip velocity is then;

$$\text{VELOCITY}\;=\;\sqrt{Vx^{2}+Vy^{2}+Vz^{2}}$$

Differences between test conditions were analyzed using paired T-test.

## III. Results (Clinical Testing)

Data was available from 11 subjects for the pick and place task, and 10 subjects for the motion capture experiment. Two subjects were excluded from the motion capture analysis due to technical difficulties. One subject was excluded from pick and place tasks (time constraints).

**Fig. 3.**
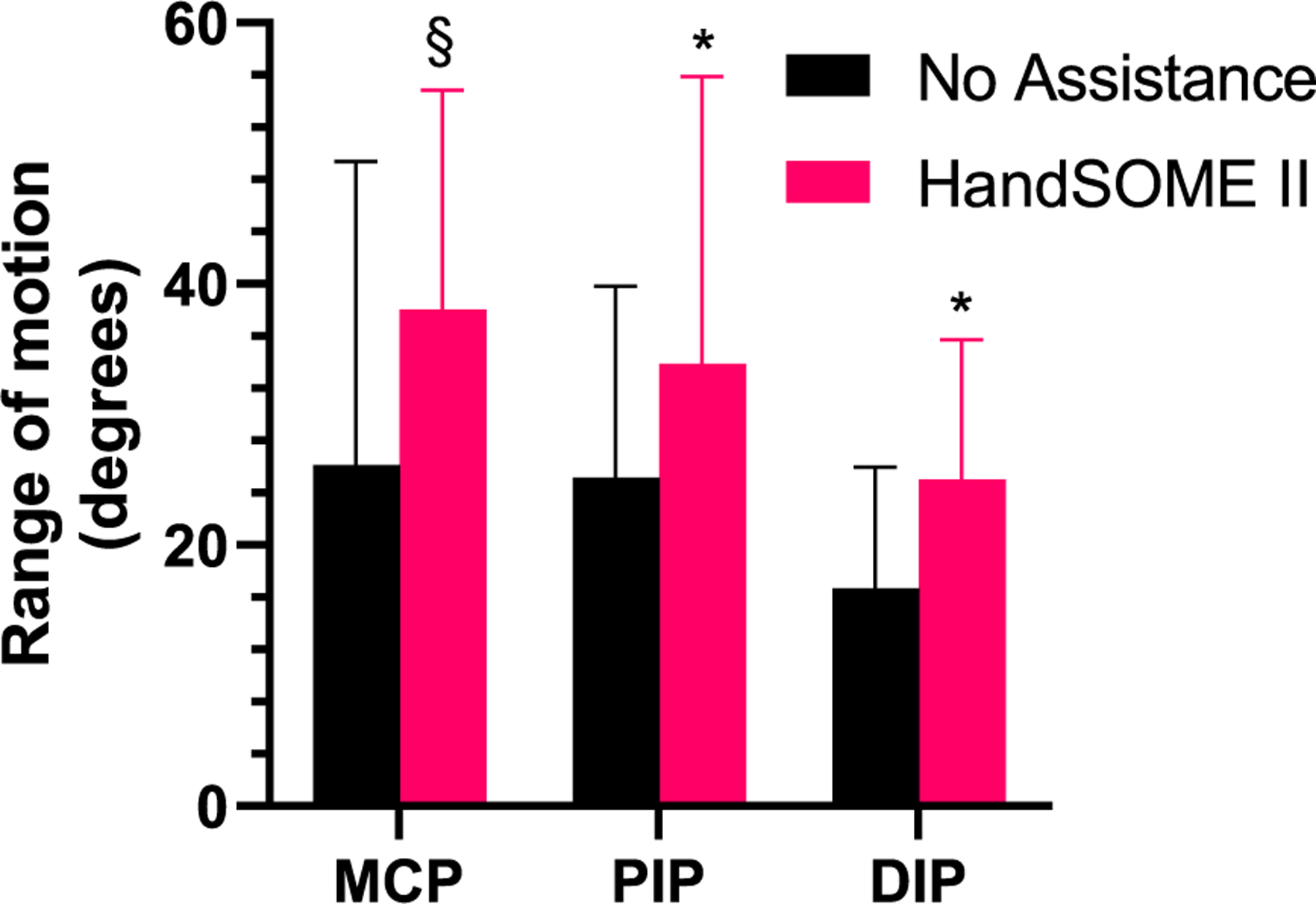
Max range of motion at the index finger joints with and without the device. Error bars indicate STD (* p ≤ 0.05, §p<0.1).

Kinematics were compared when subjects were using the device versus the bare hand. HandSOME II increased ROM significantly in the index finger PIP (p=0.0104) and DIP (p=0.026), with improvements at the MCP approaching significance (p=0.056). Mean gains were 12±17° at the MCP, 11±11° at the PIP, and 8±10° at the DIP (Fig. 3).

**Fig. 4.**
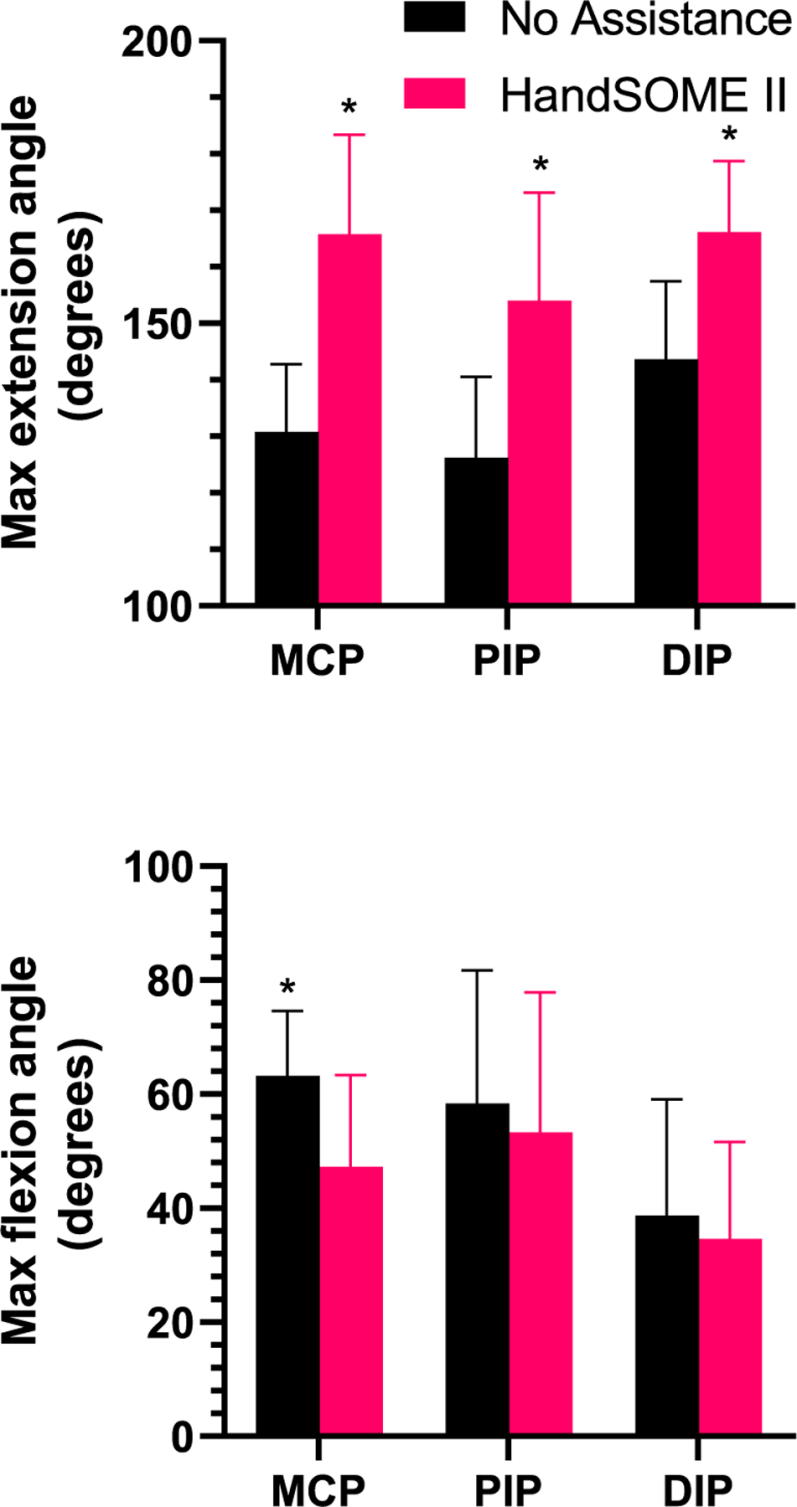
(Top) Max extension angle and (Bottom) max flexion angle at the index finger joints with and without the device. For max extension angle, 180 degrees signifies a flat extended hand. For max flexion angle, 0 degrees signifies a flat extended hand. Error bars indicate STD (* p ≤ 0.05). Max extension angle based on subgroup analysis of subjects with more than 10 deg of deficit at each joint.

The max extension angle of MCP, PIP, and DIP index finger joints was calculated across subjects. The max extension angle increased in the MCP with significant mean gains of 28±20° (p=0.002, n=10), PIP mean gains of 14±20° (p=0.06, n=10), DIP mean gains of 12±18° (p=0.052, n=10). In some cases, hyperextension without the device was prevented by the device (Fig. 2(c)). This decreases maximum extension but is a positive change. Additionally, in joints with little or no extension deficit, no rubber bands were added and no increase in maximum extension was expected. When only considering joints with more than 10 degrees of initial extension deficit, the maximum extension angle increased significantly at the MCP (p<0.001, n=8), PIP (p=0.013, n=6) and DIP (p=0.016, n=6). Mean gains were 35±15° at the MCP, 28±18° at the PIP, and 23±15° at the DIP (Fig. 4; Top).

After donning HandSOME II, the max index finger flexion angle decreased across subjects (Fig. 4; Bottom), with declines of −16 ±14° at the MCP (p=0.005, n=10), −5 ±20° at the PIP (p=0.445, n=10), and −4±18° at the DIP (p=0.49, n=10). Differences were only signifnicant at the MCP joint.

**Fig. 5.**
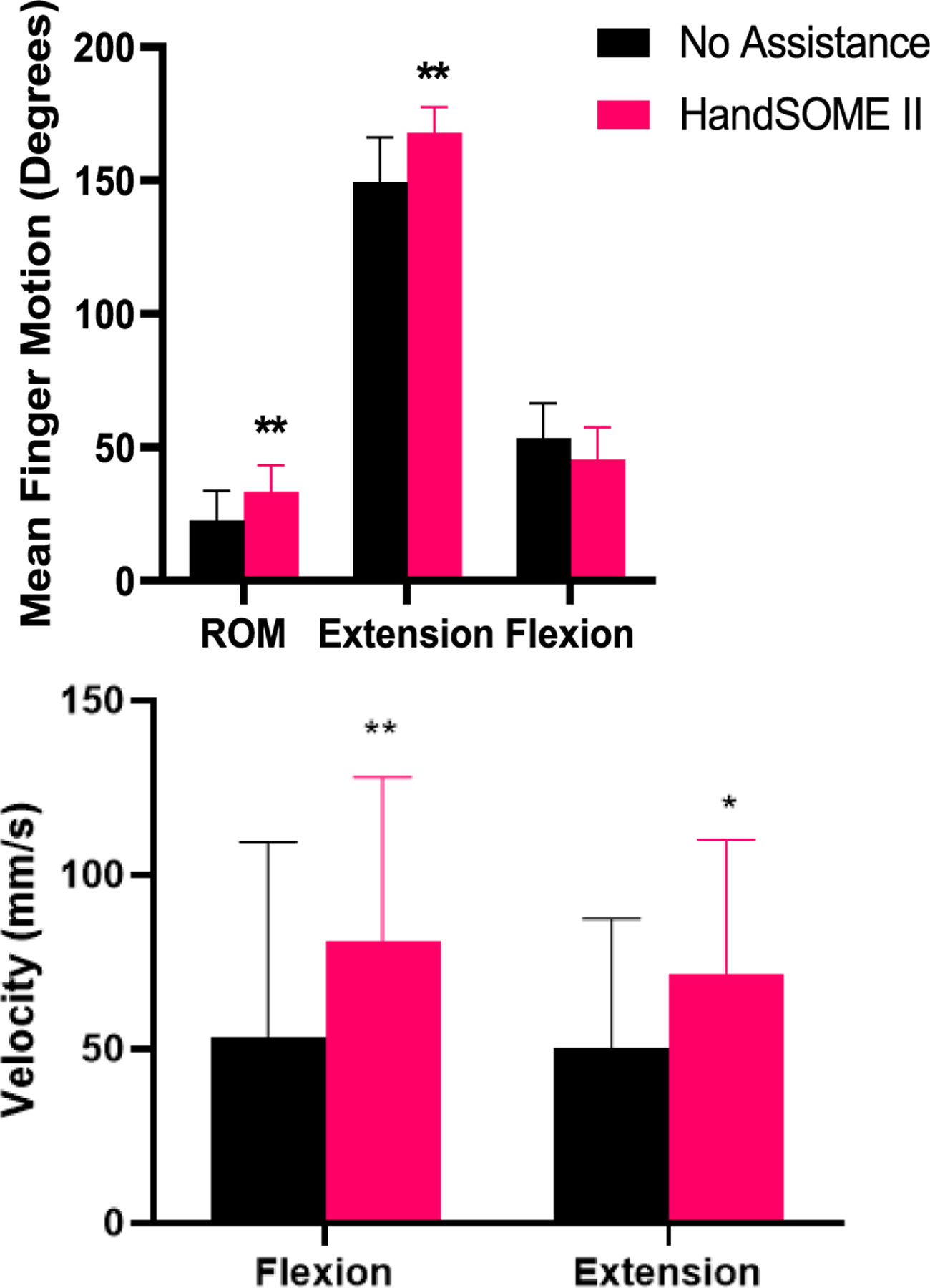
(Top) Compound index finger angle. (Bottom) Index finger tip velocity. Error bars indicate STD (* p ≤ 0.05, ** p<0.01).

Analysis of compound index finger movement showed significant increases in ROM_FINGER_ (p = 0.0022) and EXT_FINGER_ (p = 0.0029) while wearing the HandSOME II across all subjects (n=10) (Fig. 5, Top). Additionally, HandSOME II significantly increased flexion (p=0.0048) and extension VELOCITY_FINGER_ (p=0.012, Fig. 5; Bottom) compared to bare hand.

**Fig. 6.**
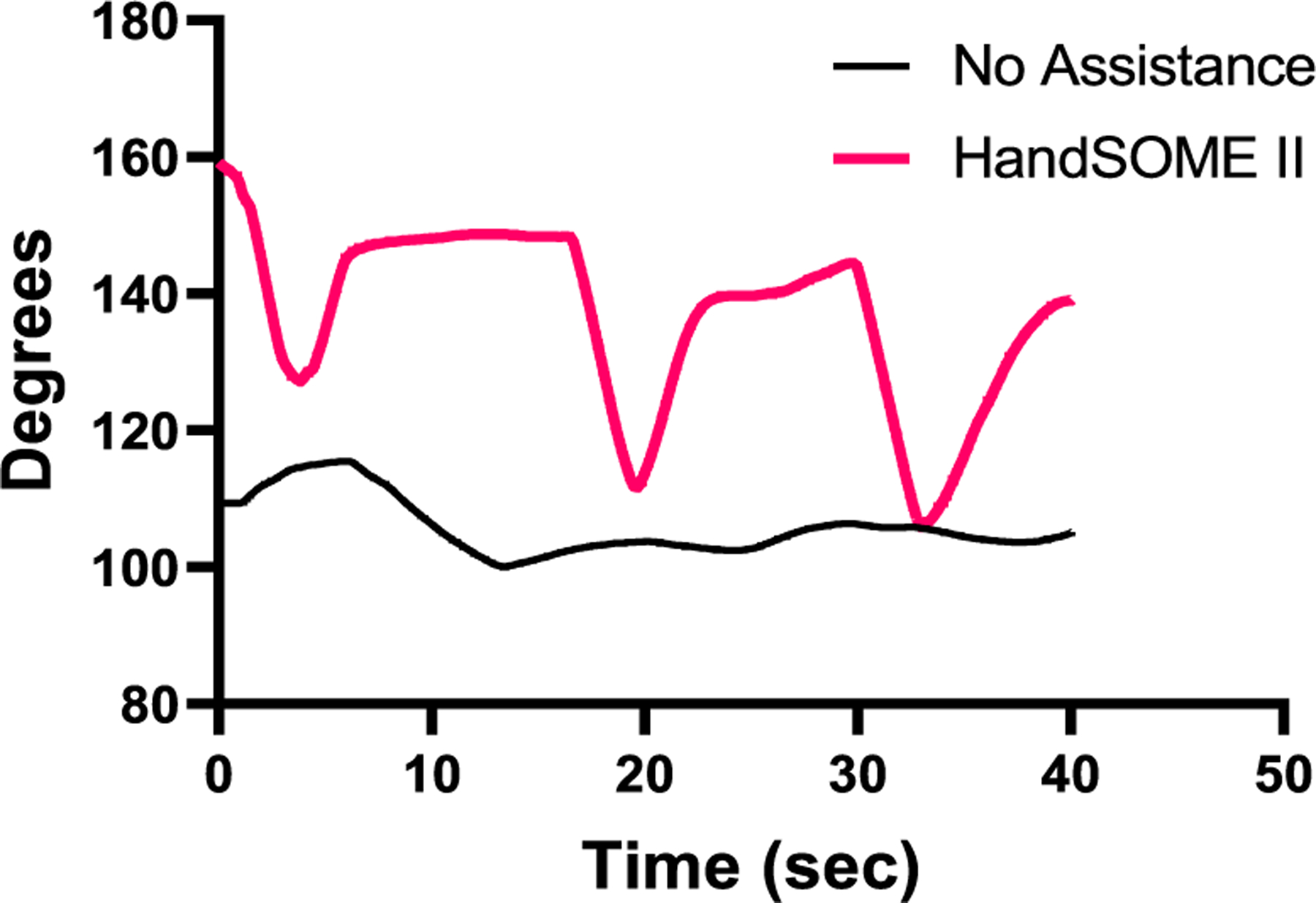
Individual subjects PIP kinematics with and without the HandSOME II device. 180° represents full extension.

**Fig. 7.**
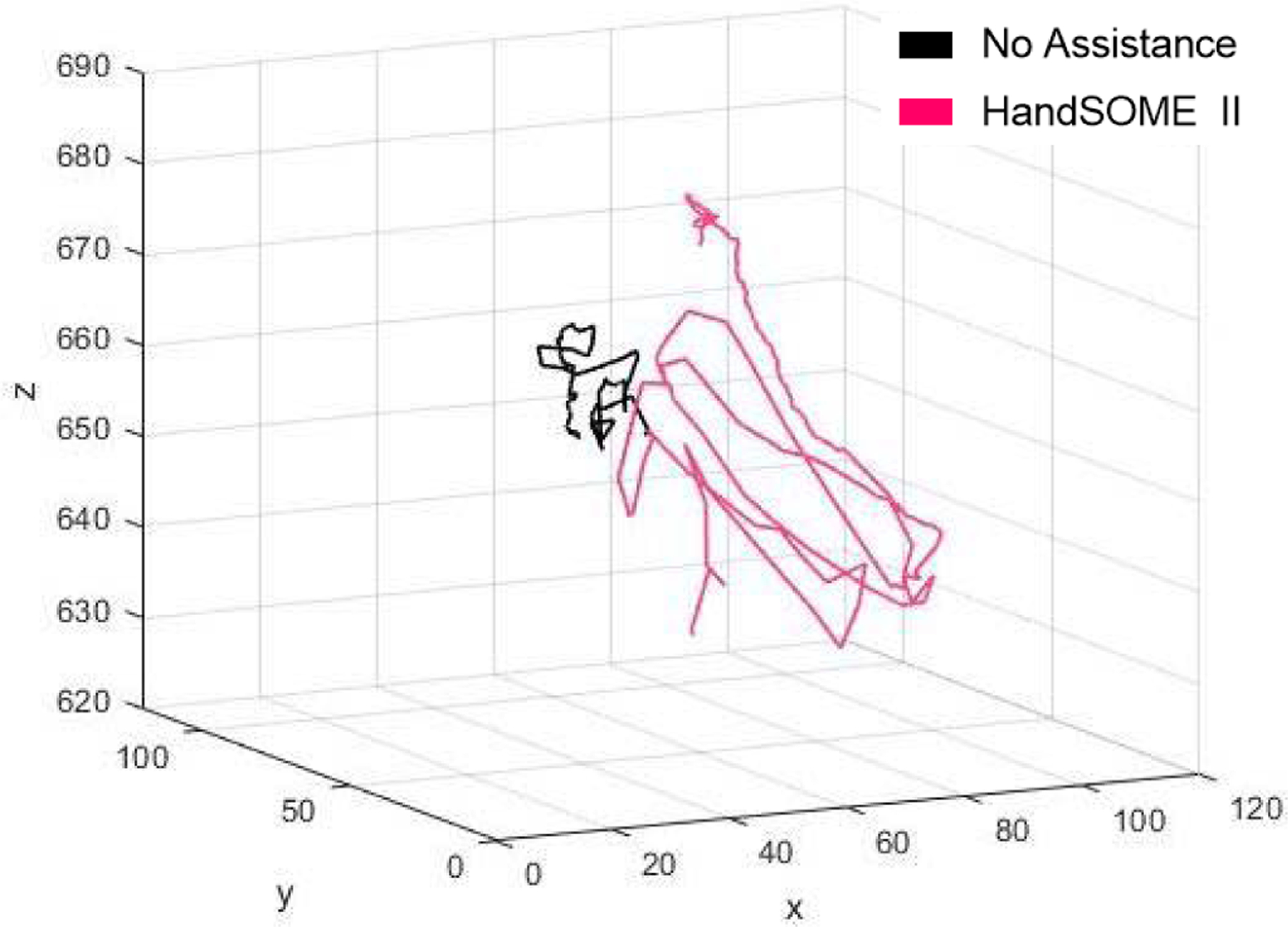
Individual subjects finger tip trajectory kinematics with and without the HandSOME II device. Axis units are in mm.

Fig. 6 shows kinematic index finger patterns from one subject who had large improvements in range of motion after donning HandSOME II. Figure 6 also suggests that velocity at the PIP joint was also increased for this subject when wearing the device. Fig. 7 depicts the index fingertip trajectory of this subject, again showing a larger ROM while wearing the HandSOME II.

**Fig. 8.**
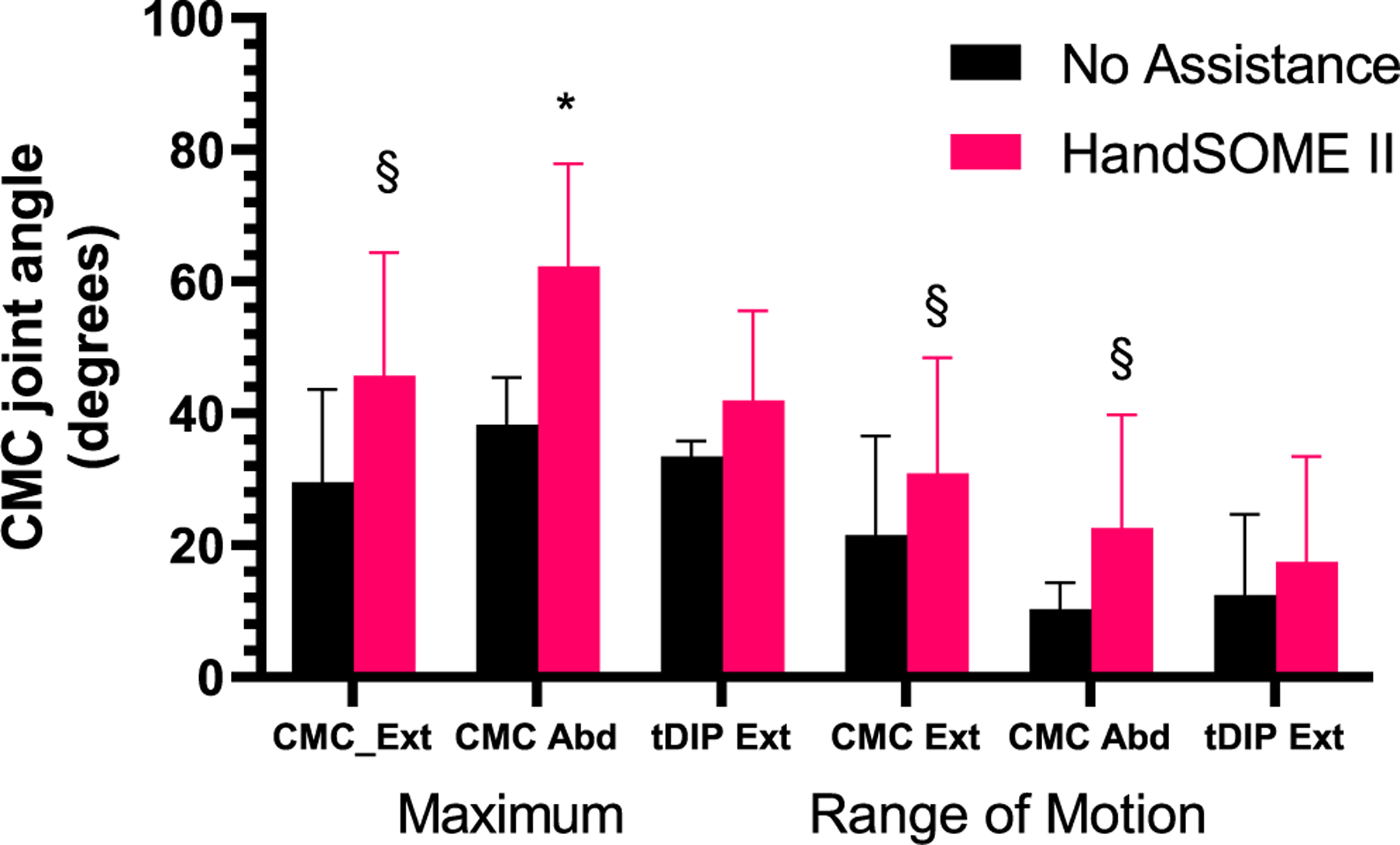
Joint angle comparison of thumb with and without the device averaged across the 7 subjects (* p ≤ 0.05, §p<0.1).

**Fig. 9.**
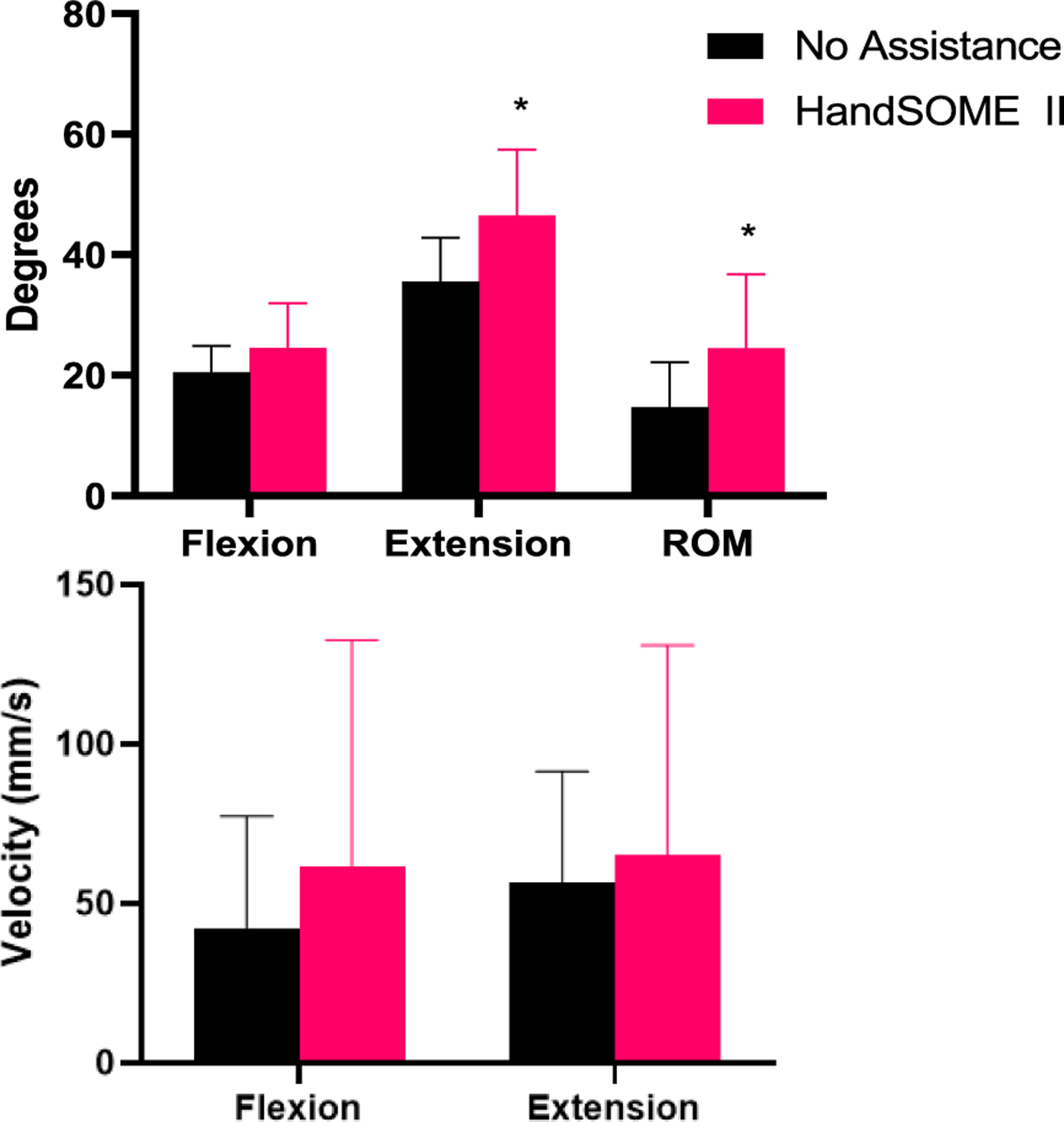
(Top) Compound thumb angle (Bottom) Thumb tip velocity when wearing HandSOME II. Error bars indicate STD. (* p ≤ 0.05, ** p<0.01).

**Fig. 10.**
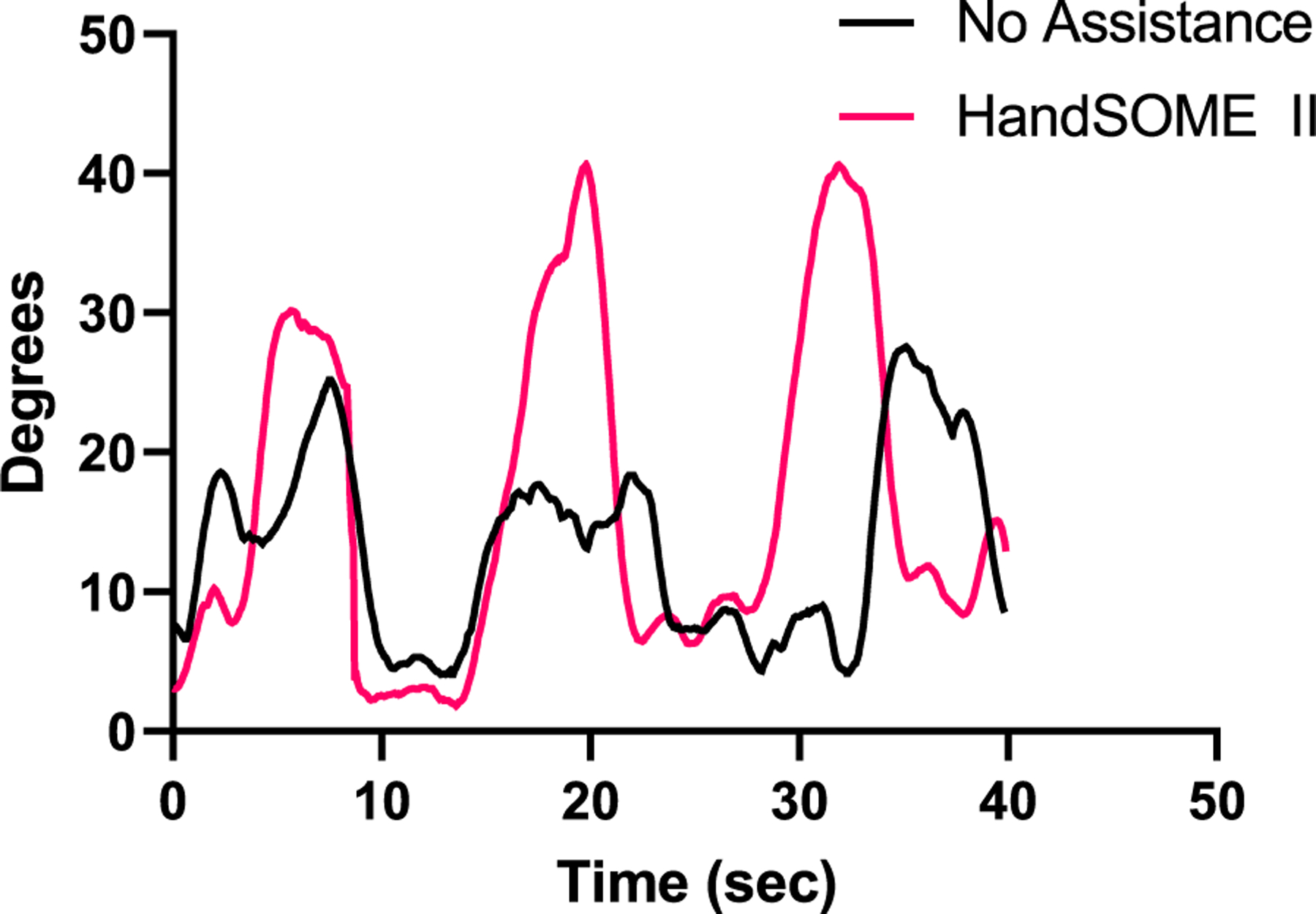
Individual subjects CMC extension kinematics with and without the HandSOME II device.

**Fig. 11.**
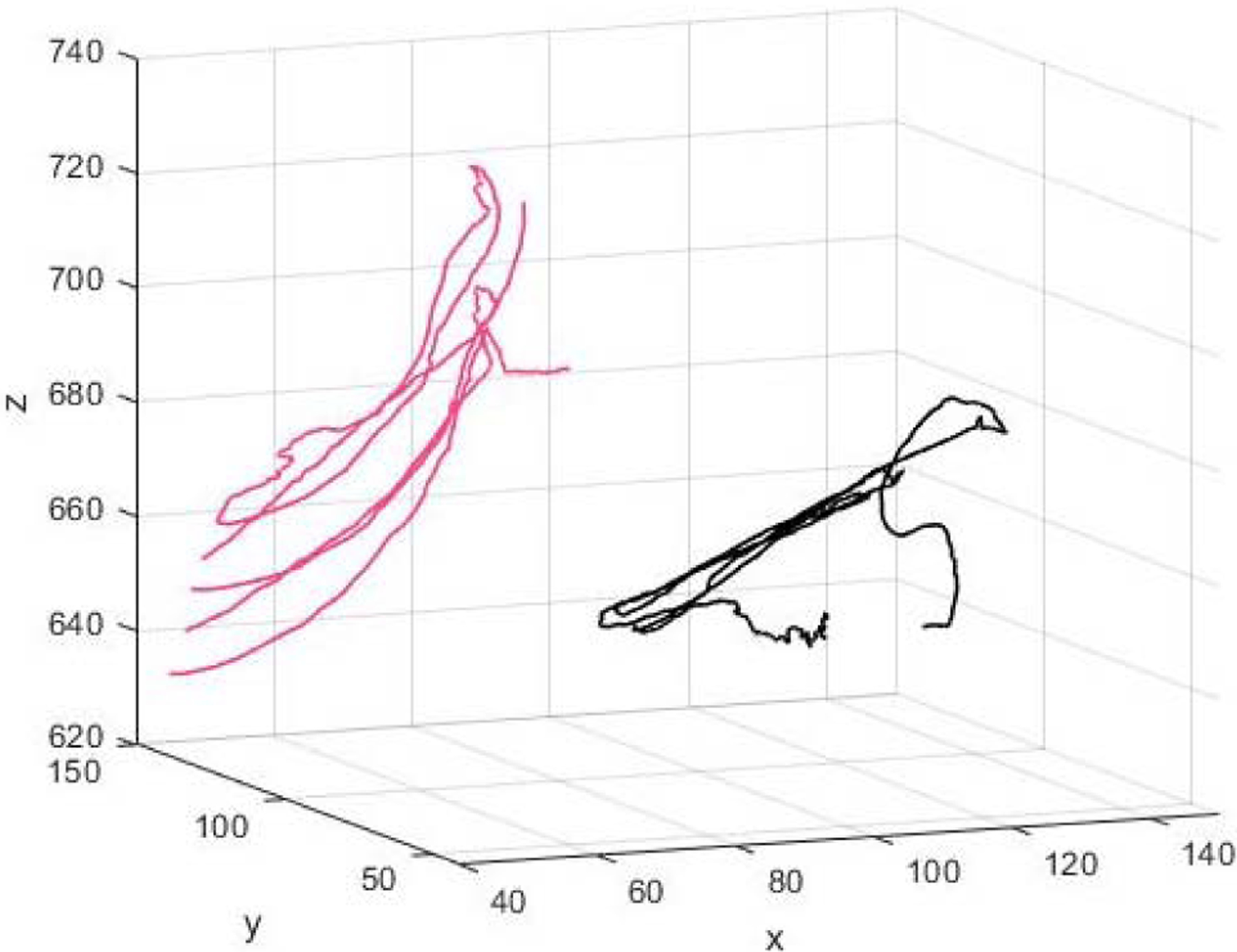
Individual subjects thumb tip trajectory kinematics with (red) and without (black) the HandSOME II device. Axis units are in mm.

Thumb kinematic data was available from 7 subjects. For CMC flexion/extension, use of HandSOME II produced mean gains in maximum extension (14.18±20.12°, p=0.078) and range of motion (8.05±11.8°, p=0.1) (Fig. 8). For CMC abduction/adduction, use of HandSOME II produced mean gains in maximum abduction (23.97±14.58°, p=0.0048). There were no significant improvements in CMC abduction ROM, tDIP maximum extension or tDIP ROM when wearing the HandSOME II device. When considering total thumb motion, HandSOME II signficantly improved EXT_THUMB_ (14.73±11.49°, p=0.0146; Fig. 9; Top) and ROM_THUMB_ (9.9±9°, p= 0.03; Fig. 9; Top). There was also an improvement in VELOCITY_THUMB_ during opening and closing movements but this was not significant (Fig. 9; Bottom). Fig. 10 shows CMC extension kinematics from one subject who had improvements in range of motion after donning HandSOME II. Figure 10 also suggests extension/flexion velocity at the CMC joint was also increased for this subject when wearing the device. Figure 11 depicts the thumbtip trajectory of this subject, again showing a larger ROM while wearing the HandSOME II.

**Fig. 12.**
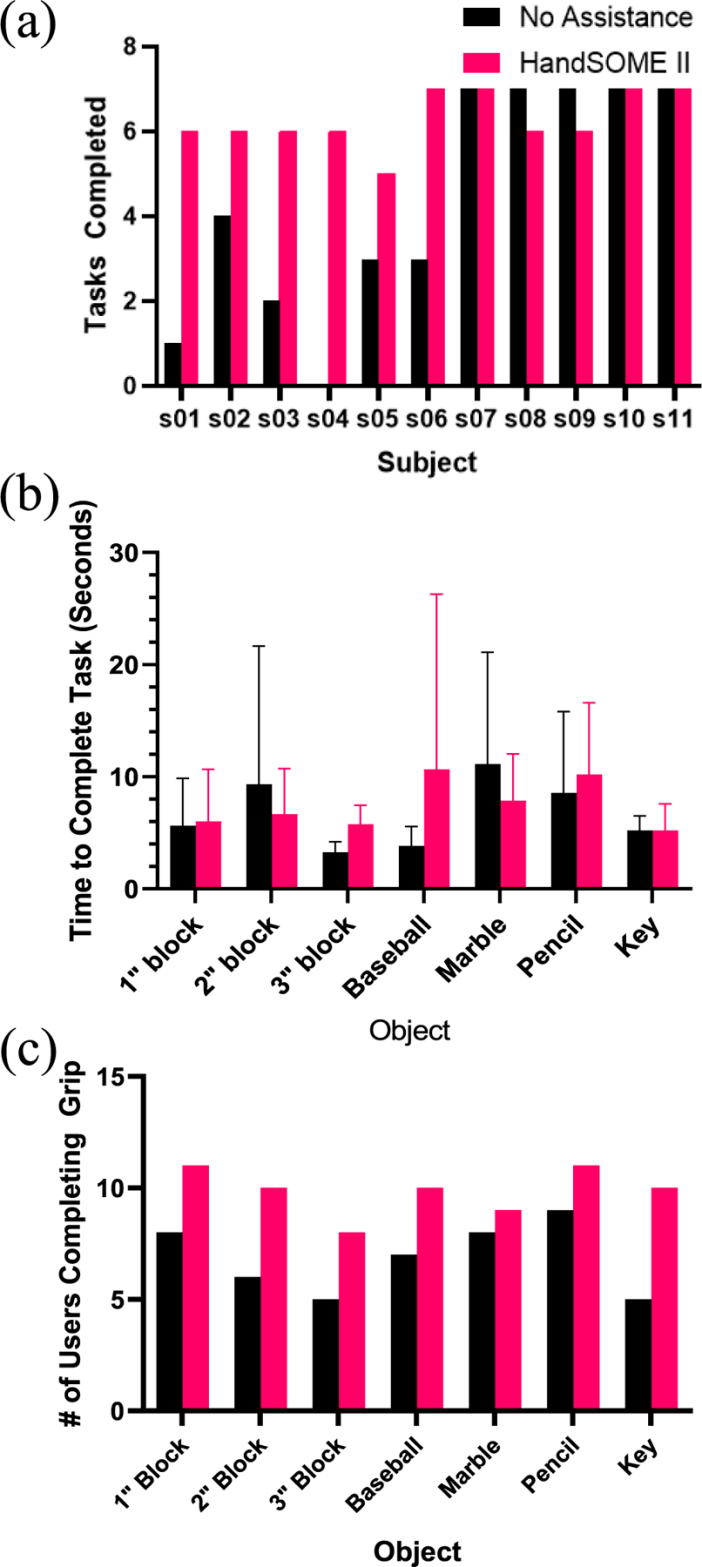
(a) Results for number of successful “pick and place” tasks completed by each subject with and without the assitive device. (b) Movement time data of objects that subjects could grasp and release. (c) Objects users were able to grip barehanded and with device.

In Fig. 12(a), we compared the number of tasks completed by each subject with and without the device. Across all subjects, use of HandSOME II device increased the number of tasks that could be completed (p=0.0302). Among the eleven subjects who attempted this test, six of them were unable to complete all of the functional tasks unassisted. In these 6 subjects, 13 tasks were completed without assistance, while 36 tasks were completed when wearing HandSOME II. Task success was not particular to a specific object, with each subject benefitting uniquely to task completion with the device. The other five subjects could complete all of the tasks without assistance. In this case, the device did not interfere with task performance in 3 of these 5 subjects, and all of the tasks were also completed when wearing HandSOME II. The device seemed to interfere with performance of the other 2 subjects who each performed one less task when using the device (gripping 3” block and marble).

We analyzed the movement time data of each object that subjects could successfully grasp and release, with and without HandSOME II (Fig. 12(b)). Tasks were omitted from comparison if the subject failed to complete the task with or without the device. While in previous results we proved the completion of more tasks with the device, these results showed that the device did not significantly slow down the task movement time. Without the device subjects took an average time of 6.7±3.0 secs, and with the device subjects took 7.5±2.1 secs. In terms of the number of subjects who completed each pick and place task, there were improvements in all 7 objects, with the smallest improvement in the marble and the largest improvement in the key (Fig. 12(c)).

There were 3 cases (3” block, baseball, marble) where the object was successfully picked up with the bare hand, but the subject failed when wearing the HandSOME II. In the case of the 2 large objects (3” block and baseball), max extension and ROM increased in both subjects, but max flexion at the MCP decreased, which might indicate inadequate grip force. Grip force on one of these two subjects decreased by 10 lbs when wearing the device, although max grip force still seemed adequate at 20 lbs. For the case of the marble, the subject’s index finger flexion max decreased at 3 joints and grip force dropped by 5 lbs, which might have caused the failure for this small object.

The average grip force for 10 subjects (two subject were not tested due to time constraints) was 19.7±10.3 lbs when tested without the device, and 17.5±10.3 lbs while wearing the device. Thus, average grip force decreased by only 11.2% while wearing the device and this difference was not significant (p=0.398).

**Fig. 13.**
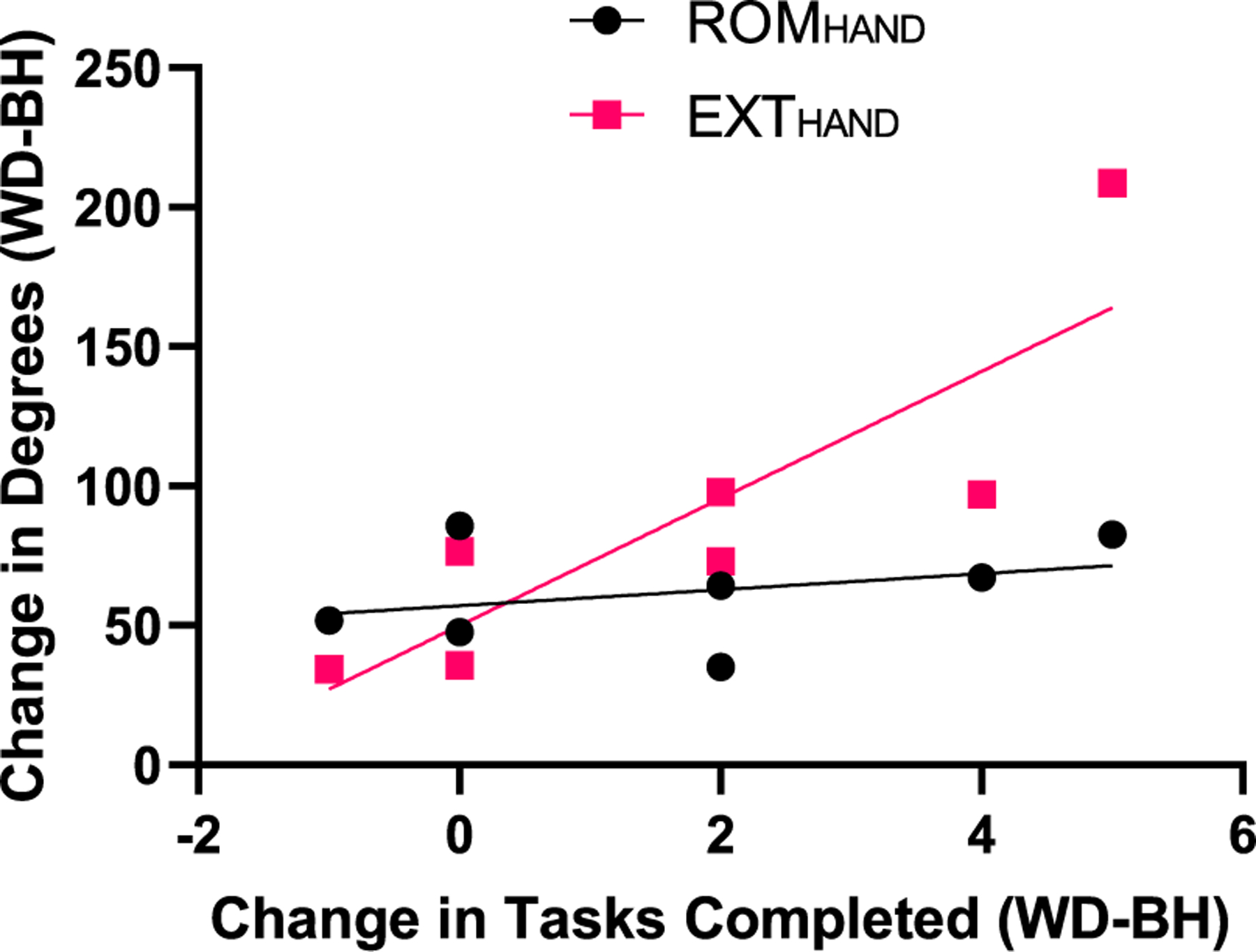
Linear regression analysis of change in degrees of hand opening while donning HandSOME II vs. change in the number of tasks completed while donning HandSOME II (ROM_HAND_ R^2^ = 0.11 and EXT_HAND_ R^2^ = 0.74). Hand opening was avilable from 7 subjects only, due to loss of thumb data on 4 subjects.

Linear regression analysis showed a positive relationship between EXT_FINGER_ and the number of objects subjects were able to pick up (N=20, p = 0.037, R^2^ = 0.25). There was also a positive relationship between ROM_FINGER_ and the number of objects subjects were able to pick up (N=20, p=0.035, R^2^ = 0.25). Both ROM_HAND_ and EXT_HAND_ significantly increased while using the HandSOME II (p = 0.0001 and p=0.0071 respectively). There was a significant relationship between subjects change in EXT_HAND_ and change in number of tasks they could complete after donning the HandSOME II (p=0.013). Fig. 13 shows that as subjects increased EXT_HAND_ by wearing HandSOME II; the number of objects they were able to pick up increased (R^2^ = 0.74).

## IV. Discussion

Our results demonstrate that chronic stroke patients can improve function of the affected hand immediately by donning the HandSOME II device. Specifically, we showed mean gains in max extension angles and ROM in all of the joints examined, and gains were significant at most of the joints (index finger MCP, PIP and DIP, thumb CMC, and thumb DIP). Furthermore, we showed these improvements in joint range of motion translated into functional improvements. This was further demonstrated by 6 subjects who could complete more grasp and release tasks while wearing the HandSOME II device. These 6 subjects were also able to grasp and release larger objects while wearing the HandSOME II device, which is consistent with the improvement in max extension found in the biomechanical evaluations. Grip force decrease was not significant, which is important as max grip force is also impaired in this population. There were 3 instances of subjects failing in a grasp and release task with the device even though they could complete the task previously. Comparison to the kinematics suggest this interference might have been due to decreased maximum flexion angles and grip force.

While use of HandSOME II device decreased index finger MCP maximum flexion angle, total index finger ROM and total hand ROM increased significantly. Moreover, index finger flexion velocity increased while wearing the HandSOME II. While this is a positive result, considering the HandSOME provides extension only assistance, improvements in index finger flexion velocity were not expected. However these results could be explained by the significant increase in ROM that subjects experienced when using the HandSOME II, allowing a longer duration to accelerate the index finger. Importantly, wearing the device did not impede the ability to complete different grasp patterns or pick up smaller objects. All subjects were able to pick up a 1” block and pencil when wearing the device. A device that supports multiple grips and manipulation of objects of varying sizes is essential for task specific training, which we have achieved with the HandSOME II. Task specific training, with and without assistive devices, has been shown to elicit neural plasticity; a potential key factor in promoting motor function gains post stroke [22]. Studies show cortical reorganization of motor maps including an increase in the size of the corresponding affected hand/arm representations in the ipsilesional hemisphere following task-specific training [23]. A recent study investigated neural plasticity of chronic stroke patients during device-assisted task-specific upper limb therapy [24]. They reported improvements in the Box and Blocks Test and active ROM post intervention corresponded with a shift in cortical activity during hand opening from the contralesional to the ipsilesional hemisphere, suggesting an increased use of the corticospinal tract as opposed to the corticobulbar tract, which has inadequate innervation of hand extensor muscles [25]. In moderate-severely impaired patients, task-specific training and neural plasticity might be facilitated by use of HandSOME II, which enables expanded ability to successfully perform grasping and releasing of a variety of objects. Future studies are required to determine any long term benefits of extended use of HandSOME II in stroke patients.

On average, subjects lost 2.2 lbs. of grip force while wearing the device. In previous work, we showed that the torque profiles of the spring assistance provided by the device drop to zero when the device was fully closed. Thus, loss of grip force is attributed to extensor forces from the springs. However, this only represented an 11.2% decrease overall and two patients increased their grip force which we attribute to improved range of motion and grip alignment with the dynamometer when wearing HandSOME II. It is not possible to know if this average decrease in grip force would hinder the patients hand function, but if it was a major factor, we would have expected significant decrease in performance in the pick and place tasks. We did observe a decrease in performance with HandSOME II in 3 cases, which might have been due to decreased max grip force; however we did observe 24 cases of an object being successfully picked and placed with the device when the subject failed with the bare hand. We measured grip force with a Jamar dynamometer. In future studies, we plan to also measure palmer pinch force as decreases in peak force might be larger in more flexed hand postures.

Passive spring-based designs, such as HandSOME II, are more compact and lighter than actuated devices. Furthermore, without the need for air compressors [26], [27], electric motors [28]–[31], this wearable device has advantages for take-home use, allowing subjects the option to use the device during activities of daily living. A limitation of our passive device is that assistance is only provided to extension movements. This excludes any patients with severe weakness in the flexor muscles. Patients must also be able to relax the flexor muscles after a grasp, in order for the springs to move the fingers into extension. In these cases, active devices such as HandMATE [31], Maestro [32], X-Glove [14] might be more effective for hand rehabilitation by providing assistive forces to close and open the hand. Another design limitation of our current device is the isolated assistance for the hand with no assistance for the rest of the upper limb. Severe proximal weakness could compromise effectiveness of HandSOME II. In repetitive task training, we envision the device would be used at the discretion of the therapist, who might incorporate a wrist brace or a gravity compensation arm support to facilitate the training. In some patients with good proximal control, these supports might not be needed, and the HandSOME could be used alone during training. We are also developing a wearable gravity compensation device for supporting patients with proximal weakness [33]. Using the device for an extended period may fatigue the subject, especially finger flexors which are contracting against the rubber bands. Our testing was not extensive enough to observe fatigue, however a prior study with HandSOME I found subjects tolerated 1.5 hours/day of wear time with the device. In that study, the mean number of movements per day was 448±651.

Some limitations in our study design should be noted. The grasp types tested should be expanded into include items from the grip category of the ARAT. There might have been motor learning in the first set of trials of the pick and place tasks without the exoskeleton, which resulted in better performance in the 2^nd^ set of trials with the exoskeleton. We tried to mitigate any motor learning by allowing no practice and only a 30 sec period for each object, and using common objects used in daily activities. The order was chosen because we felt that fatigue would be a problem in the 2nd set of trials, and we wanted this to be a disadvantage for the exoskeleton. Patients were allowed to use their unaffected arm to support the affected arm in all trials, both with and without the exoskeleton. It is possible the subjects provided more gravity assistance in the trials with the device than without. We have no way of measuring this, but we have no reason to believe there was systematic bias on the part of the patients.

The SaeboGlove is a commercial product that has similarities with our device in that both are powered by rubber bands. We are not aware of published reports on the performance of the SaeboGlove, however we believe the main differences between the SaeboGlove and our device are the torque profiles. The SaeboGlove uses rubber bands along the surface of the glove, similar to exotendon approaches. HandSOME II applies torque to the joints through an exoskeleton with centers of rotation aligned with the finger joints. This allows application of force normal to the finger segments and increased comfort, with no compressive forces through the joint. The exoskeleton also enables customizable torque vs. angle profiles using optional wrapping pins.

In prior work with HandSOME I, stroke subjects performed reach and grasp task practice at home for 1.5 hours/day, 5 days per week, for 4 weeks [18]. Significant gains were found in measures of impairment and functional outcome. The subjects who complied with the target dosage achieved the largest gains on clinical scales, which were also accompanied by improved range of motion and increased digit extension. However, gains were lost at the 3-month follow-up. HandSOME II provides customizable assistance via 11 different spring settings in contrast to the 1 DOF HandSOME I. Therefore, we expect greater gains when training with HandSOME II, which allows a much wider range of grasping patterns and more subject-specific customization options of assistance levels compared with HandSOME I. However, for practical reasons, it might be that some patients would benefit more from HandSOME I and others HandSOME II. For example, patients who are unable to don or use HandSOME II effectively, might be able to use HandSOME I, which is easier to don, and easier to fit and adjust. We did not test donning ability this in this study, however in subsequent work, most patients were able to don/doff the device independently with guidance and training from a therapist. We would hope that after a period of practice with HandSOME I, patients could transition to the HandSOME II to work on more complex grasp patterns.

## V. Conclusion

We have developed an exoskeleton that allows movement at 15 hand DOF. Eleven separate elastic elements can be added to customize the extension assistance for individual subjects. In testing with chronic stroke subjects, both range of motion and max extension angles were significantly increased when wearing the device. While it is not surprising that applied extension torque would increase max extension angles, the ability to apply torque profiles that decrease with increasing flexion allowed the ROM to also increase and the grip force to only decrease by 11.2%. The high DOF of this device will theoretically allow a large range of grasp tasks and individual finger movements. Use of the device would allow practice of object manipulation tasks even in subjects with poor hand extension ability and may be more effective than unassisted task practice for therapeutic gains. Future work will consist of extended independent home training with the device.

## Supplementary Material

supp1-3110201
