# Supplementary material for: Clinical Test of a Wearable, High DOF, Spring Powered Hand Exoskeleton (HandSOME II)
Source: IEEE Trans Neural Syst Rehabil Eng. Author manuscript; Available in PMC 2021 Sep 24. (PMC8462990; doi:10.1109/TNSRE.2021.3110201)
Supplement: supp1-3110201 [file NIHMS1741061-supplement-supp1-3110201.docx]

Supplementary Table 1. Patient characteristics and experimental results

| **Subject** | **Age**  **(Yrs)** | **Gender** | **Time Post Stroke**  **(Months)** | **FMA** | **Finger ROM**  **(ROM_FINGER_)°** | | **Finger Max Extension (EXT_FINGER_) °** | | **Finger Flexion**  **(FLEX_FINGER_) °** | | **# Tasks Completed (BH - WD)** |
| --- | --- | --- | --- | --- | --- | --- | --- | --- | --- | --- | --- |
|  |  |  |  |  | **BH** | **WD** | **BH** | **WD** | **BH** | **WD** |  |
| 1 | 38 | M | 63 | 31 | 24 | 24 | 129.3 | 149.7 | 105.3 | 125.7 | 1 - 6 |
| 2 | 64 | F | 28 | 34 | 26.3 | 43.7 | 142.7 | 166.3 | 116.3 | 122.7 | 4 – 6 |
| 3 | 59 | M | 58 | 43 | 36.3 | 36 | 179 | 180.7 | 142.7 | 144.7 | 2 – 6 |
| 4 | 39 | M | 37 | 19 | 8 | 25.3 | 132.3 | 182 | 124.3 | 156.7 | 0 – 6 |
| 5 | 66 | F | 28 | 23 | 12 | 16 | 153.3 | 162.7 | 141.3 | 146.7 | 3 – 5 |
| 6 | 58 | M | 12 | 29 | N/A | N/A | N/A | N/A | N/A | N/A | 3 – 7 |
| 7 | 65 | M | 47 | 47 | 34 | 44 | 156.7 | 165 | 122.7 | 121 | 7 - 7 |
| 8 | 73 | M | 20 | 36 | 21.7 | 42.7 | 151 | 167 | 129.3 | 124.3 | 7 – 6 |
| 9 | 38 | M | 53 | 47 | 35 | 41.3 | 151.3 | 172 | 116.3 | 130.7 | 7 – 6 |
| 10 | 75 | F | 17 | 34 | N/A | N/A | N/A | N/A | N/A | N/A | 7 – 7 |
| 11 | 61 | F | 7 | 20 | 24 | 34.7 | 169 | 172.3 | 145 | 137.7 | 7 – 7 |
| 12 | 73 | F | 21 | 15 | 5.3 | 15.3 | 153.3 | 162.7 | 122.3 | 139.3 | N/A |

FMA=Fugl-Meyer assessment

BH = Bare Hand

WD = With Device
